# Case report: Use of three-dimensional technology in criss-cross heart with double outlet right ventricle

**DOI:** 10.3389/fcvm.2023.1172104

**Published:** 2023-05-05

**Authors:** Ailixiati Alifu, Haifan Wang, Yuntian Su, Renwei Chen

**Affiliations:** Department of Cardiothoracic Surgery, Hainan Women and Children's Medical Center, Haikou, China

**Keywords:** congenital cardiac malformation, crisscross heart, three-dimensional printed heart model, surgical planning, case report

## Abstract

**Background:**

In this case report, we utilized a three-dimensional printing model to replicate the complex anatomy of a criss-cross heart with double outlet right ventricle—an extremely rare congenital cardiac abnormality. This approach facilitated our understanding of the patient's unique condition and enabled us to plan the surgical procedure with greater precision.

**Case presentation:**

Our department received a 13-year-old female patient who presented with a pronounced heart murmur and a decrease in exercise capacity. Subsequent two-dimensional imaging revealed the presence of a criss-cross heart with double outlet right ventricle—an intricate and uncommon cardiac malformation that poses challenges for accurate visualization through conventional two-dimensional modalities. To address this challenge, we constructed and printed a three-dimensional model using computed tomography data, which enabled us to visualize and understand the complex intracardiac structures and plan surgical interventions with greater precision. Using this approach, we successfully performed a right ventricular double outlet repair, and the patient made a full recovery following the procedure.

**Conclusion:**

The criss-cross heart with double outlet right ventricle constitutes a complex and uncommon cardiac anomaly that poses considerable challenges in terms of diagnosis and surgical intervention. Employing three-dimensional modeling and printing represents a promising approach, given its potential to enhance the precision and comprehensiveness of the anatomical evaluation of the heart. As a result, this method holds significant promise in facilitating accurate diagnosis, meticulous surgical planning, and ultimately improving clinical outcomes for patients affected by this condition.

## Introduction

Criss-cross heart (CCH) is an infrequent cardiac anomaly characterized by the crossing of inflow streams of both ventricles, caused by twisting of the heart along its long axis. This condition may arise due to malformations in any cardiac segment, causing disruptions in the connections between atria, ventricles, and great vessels (1). The use of three-dimensional (3D) technology is an innovative approach that offers a realistic depiction of the heart's anatomy, enabling surgeons to improve their surgical planning and communication of surgical strategies (2). In this case report, we present the use of 3D printing technology in a patient with CCH with DORV. We describe how the 3D-printed model improved the understanding of the complex cardiac anatomy and facilitated surgical planning. Our case highlights the potential of 3D printing technology in improving surgical planning and outcomes for complex congenital heart diseases, specifically criss-cross heart with DORV.

## Case presentation

**Figure 1:**
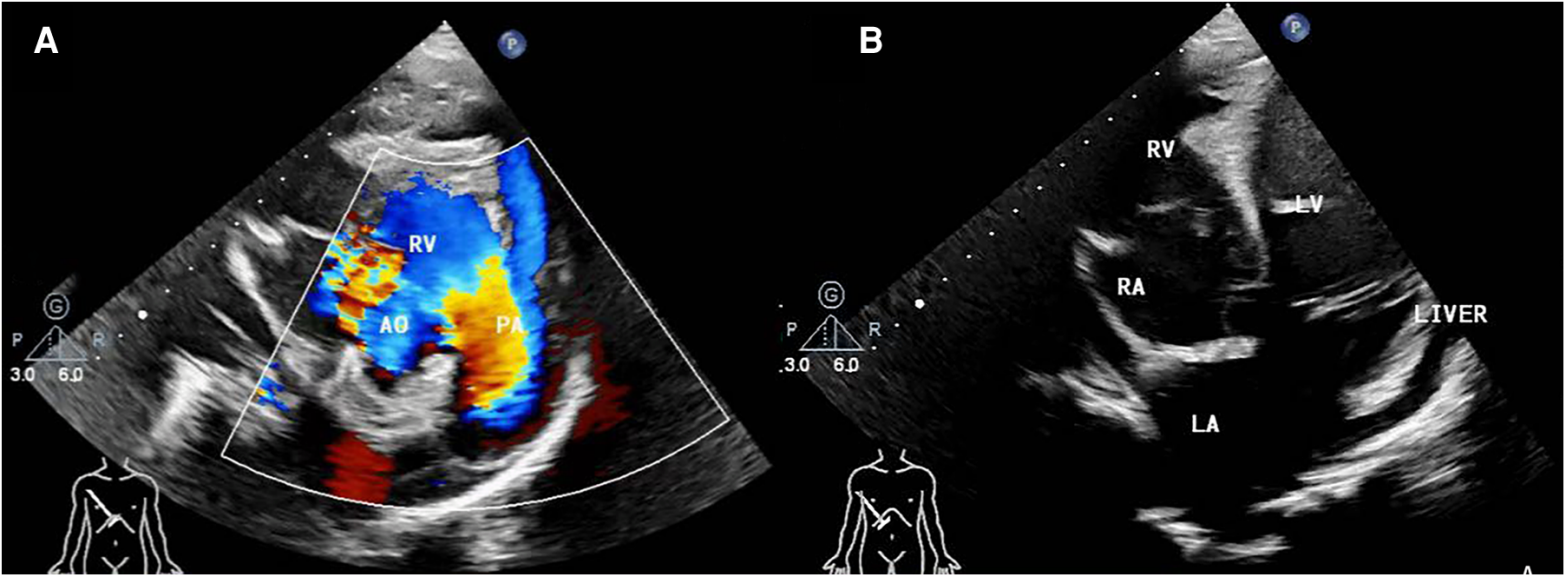
(**A**) pre-operative transthoracic echocardiogram in right parasternal short axis view, (**B**) right subcostal arch four-chamber view. AO, aorta; RV, right ventricle; PA, pulmonary artery; RA, right atria; LV, left ventricle; LA, left atria.

**Figure 2:**
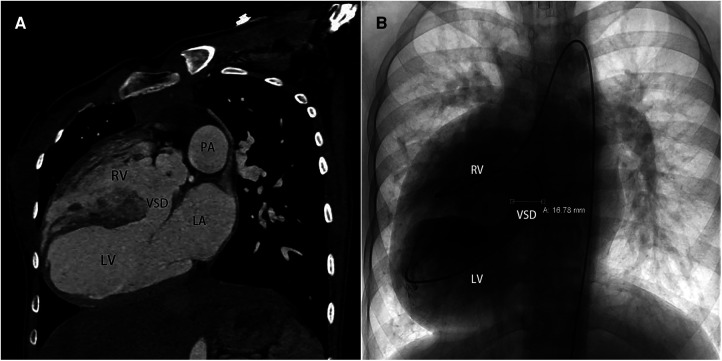
(**A**) computed tomography angiography pre-operatively in sagittal view, (**B**) cardiac catheterization in anteroposterior projection. VSD, ventricle septal defect.

**Figure 3:**
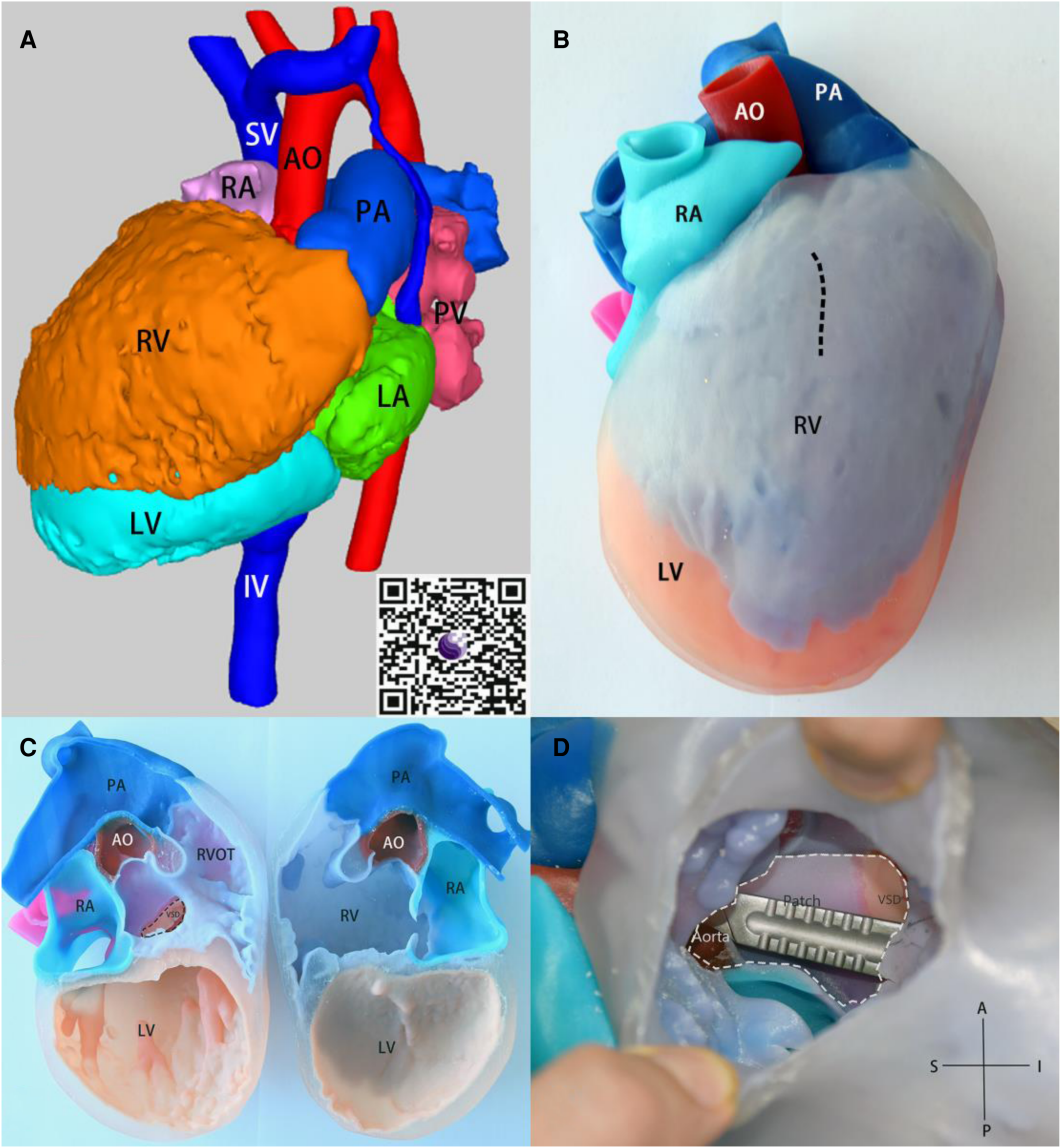
(**A**) 3D render model, obtain the 3D visualization through QR code scanning located at the lower right corner. (**B**) the 3-dimensional model in anatomical position, the pre-operative plan for cardiac incision indicated by a dashed line. (**C**) the 3D model that is divided in the middle of the coronal plane. (**D**) After incision along the dashed line in Figure **C**, the intracardiac views are shown. The white dashed line represents the patch to connect the ventricular septal defect to the aorta before surgical procedure. The Scalpel is inserted through the aorta into the ventricular septal defect to facilitate visualization of the anatomical relationship. SV, superior vena cava; IV, inferior vena cava; PV, pulmonary vein.

**Figure 4:**
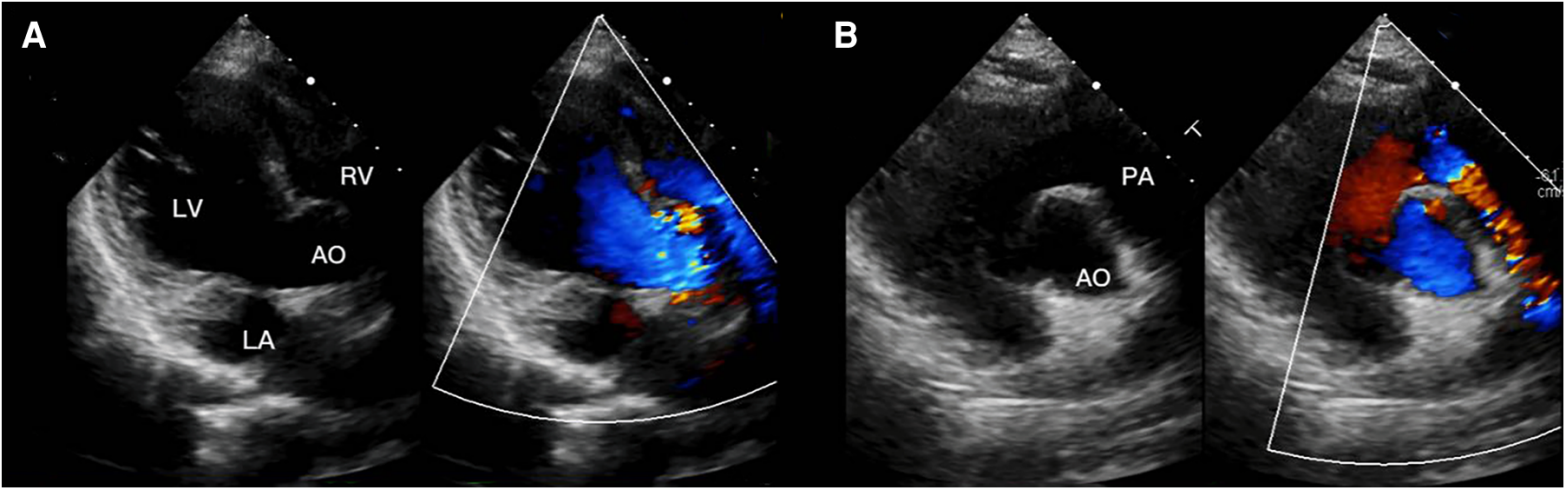
(**A**) transthoracic echocardiogram 3 months after surgery in four-chamber view of the right subcostal arch, (**B**) transthoracic echocardiogram 3 months after surgery in right parasternal short axis view.

A 13-year-old female patient, lacking a family history of cardiovascular disease, was admitted to our department with a notable heart murmur and deteriorating exercise capacity. At five months of age, the patient had been diagnosed with a multifaceted congenital heart malformation at a local hospital; however, inadequate medical technology and financial constraints prevented further diagnostic evaluation at a more sophisticated medical center. The patient thrived during her early developmental years, and the issue went unnoticed by the family until recently when her tolerance to exercise significantly declined. Upon physical examination, the child exhibited a height of 1.52 m and weight of 36 kg, with a resting heart rate of 118 beats/min and an oxygen saturation level of 97%. Notably, apical pulsation was detected in the right anterior chest, and systolic murmurs were auscultated. Following the completion of conventional imaging procedures, including transthoracic echocardiography (TTE) (Figure 1), computed tomography angiography (CTA) (Figure 2A), and cardiac catheterization (Figure 2B and Supplementary S1), we consulted with a team of imaging specialists and cardiologists to evaluate the child's complex cardiac malformation. Despite a meticulous review of the 2D images, the precise location of the ventricular septal defect (VSD) and its surrounding anatomical relationships remained unclear. Specifically, we sought to determine the feasibility of applying an unobstructed intraventricular baffle extending from the margin of the VSD to the aortic valve. For this reason, we utilized Fire Plus 3D software (Chinese Blackflame medical technology company) to perform 3D reconstruction and rendering from CTA DICOM images. The 3D model was printed using a printer (BFSLA 550Li, China) with light-cured medical resin, taking 2 days and costing 5,000 yuan (RMB). Upon careful observation of the 3D rendered imaging (Figure 3A) and thorough tactile examination of the 3D printed model (Figures 3B,C), each specialist achieved a clear understanding and unanimously concurred by declaring that “the matter is now evident.” The cardiac apex is oriented towards the right side in situs solitus. Except for the left superior vena cava, which terminates in the left atrium, the systemic and pulmonary veins drain into the respective right and left atria. The atrioventricular connections are concordant with a horizontally positioned septum. The VSD measures approximately 16 mm in diameter and is located above the mitral valve, below and to the left of the tricuspid valve, and directly below the aortic valve. Both ventricles are substantially enlarged, with the right ventricle occupying the upper right region and the left ventricle situated in the lower left quadrant, in concordance with the atrioventricular connections. An aorta is located on the right and posterior to the pulmonary trunk and primarily arises from the right ventricle. Before the surgical procedure, we strategized the surgical incision on a model to optimize visualization and access to the intracardiac structures, thereby augmenting the operative process. Based on the 3D model, it was observed that the ventricular septal defect (VSD) was near the aortic valve, while the tricuspid valve was distally positioned from the pulmonary valve (Figure 3D). Further supported by the cardiac catheterization results which indicated a pulmonary resistance of 2.19 wood and a Qp/Qs ratio (pulmonary-to-systemic flow ratio) of 7.72, it was suggested that an unobstructed intracardiac repair was a feasible option. Following the standard median sternotomy procedure, the patient underwent cannulation of the ascending aorta, right superior vena cava, and inferior vena cava to initiate cardiopulmonary bypass. The aorta was subsequently cross-clamped with antegrade cardioplegic arrest, and the left superior vena cava was ligated. Subsequently, the intracardiac structures were visualized via an incision made in the anterior wall of the right ventricle, which was consistent with the 3D-printed model. Utilizing 4-0 prolene sutures, the autologous pericardial patch was continuously sutured along the edge of the VSD and connected to the aorta. Following the successful surgery, the child's postoperative course proceeded uneventfully in the intensive care unit, with mechanical ventilation being discontinued on the fourth day. Three months after the operation, the child's postoperative transthoracic echocardiography showed excellent results (Figure 4).

## Discussion

CCH anomaly is extremely rare, accounting for less than 0.1% of all congenital heart defects. The diagnosis of CCH is based on the intersection of the axes of the ventricular entries. This condition is characterized by the mass of the ventricular wall rotating along its major axis. This promotes a change in hemodynamics characterized by crossing flows through the atrioventricular valves, resulting in the false impression that each atrium is being directed to the contralateral ventricle (1). The CCH may present with situs solitus or situs inversus and concordant or discordant atrioventricular, And ventriculoarterial connection. The atria and ventricles are related in a top-to-bottom (superior-inferior) manner, displacing the ventricular mass in the horizontal plane (3). The ventriculoarterial connection is an important factor in determining the surgical approach and prognosis for patients with CCH. In patients with discordant ventriculoarterial connection, the surgical approach typically involves switching the great arteries to their appropriate ventricles. In contrast, in patients with concordant ventriculoarterial connection, the surgical approach may involve repairing the ventricular septal defect and/or correcting the abnormal valve function. The prognosis for patients with CCH depends on the severity of the underlying heart defects and the success of the surgical intervention (4).

Several studies have highlighted the potential utility of 3D technology in medical education, surgical training, and pre-operative planning for CHD and then improve surgical outcomes and reduce costs (5, 6). Sun et al. showed the benefits of patient-specific 3D-printed models in pediatric CHD, including cases of DORV (7). Valverde et al. conducted an international multicenter study on the use of 3D-printed models for surgical planning of complex congenital heart defects, which included cases of DORV. The study found that 3D models improved surgeon confidence, reduced operating time, and decreased postoperative complications (8). According to Yoo and colleagues, 3D printing technology offers additional information for surgical planning of DORV repair compared to traditional 2D images, specifically in evaluating VSD location, the extent of the muscular infundibulum, and the distance between VSD and arterial valves. These factors are essential in assessing the feasibility of biventricular repair in DORV (9). Furthermore, Batteux et al. conducted a systematic review that concluded that 3D printing models are a valuable tool in improving surgical planning for complex congenital heart diseases (2). Zhang et al. conducted a study on 11 cases of criss-cross heart treated at Anzhen Hospital, Capital Medical University in China. The treatments included Gleen procedure for 5 cases, pulmonary artery banding for 4 cases, patent ductus arteriosus closure for 1 case, and double ventricular repair for 1 case. There were no in-hospital deaths (10).

Strengths of this report include the utilization of 3D printing technology to construct a detailed model of a CCH with DORV, which enabled the medical team to better visualize and understand the unique intracardiac structures. This approach allowed for more precise preoperative planning and accurate surgical intervention, resulting in a successful outcome for the patient. The case report also highlights the potential benefits of employing 3D modeling and printing in cases of rare and intricate cardiac anomalies. This is one of the rare reports that illustrated the anatomy of the CCH using 3D technology and assisted in preoperative planning. This case report has some limitations that must be considered. Firstly, its focus on a single patient, without a comparison group, may limit the generalizability of the findings. Additionally, the costs and time required to create a 3D print model may hinder widespread utilization of this approach. Furthermore, the use of virtual reality could aid in further illustrating the complex anatomy of CCH for diagnosis and surgical planning. Lastly, it should be noted that the 3D model employed was a hollow heart lacking valves and coronary arteries, which may impact its clinical relevance and applicability.

The present study describes a case of CCH with DORV, an extremely rare congenital cardiac abnormality that poses substantial challenges for diagnosis and surgical intervention. We report successful diagnosis and surgery through the utilization of 3D imaging and printing, yielding excellent clinical outcomes. Our findings provide compelling evidence that 3D printing technology can be employed to create highly detailed models of complex intracardiac structures, offering greater clarity and precision in anatomical evaluation and facilitating more accurate preoperative planning and surgical interventions. Nevertheless, it should be noted that the limited availability and high cost of this technology may impede its widespread adoption. Future research endeavors could further elucidate the potential benefits of 3D modeling and printing in cases of intricate and uncommon cardiac anomalies, ideally incorporating larger sample sizes and comparison groups.

## Data Availability

The original contributions presented in the study are included in the article, further inquiries can be directed to the corresponding author.
